# Natural Compounds in Plant-Based Food

**DOI:** 10.3390/foods12040857

**Published:** 2023-02-17

**Authors:** Andreas Eisenreich, Bernd Schäfer

**Affiliations:** Department of Food Safety, German Federal Institute for Risk Assessment (BfR), Max-Dohrn-Str. 8-10, 10589 Berlin, Germany

Plant-based foods include a wide range of products, such as fruits, vegetables, herbs and spices, as well as food products based on them, such as sauces, soups, or beverages. Particularly, culinary herbs and spices are used to confer characteristic flavor or coloring to those food products due to their aromatizing and color-giving properties. This, in turn, is based on the occurrence of various compounds, naturally generated as secondary plant metabolites in those herbs and spices [1]. In this context, those substances of plant origin are often regarded as harmless. However, several secondary plant metabolites, originally generated to deter pests or herbivores (e.g., phenylpropanoids and pyrrolizidine alkaloids), may also exhibit toxic properties to consumers [2,3,4].

In this Special Issue, several expert contributions addressed aspects regarding “Natural Compounds in Plant-Based Food”. These include the occurrence, toxicological relevance, and potential health benefits of different compounds, such as alkenylbenzenes, pyrrolizidine alkaloids, glucosides, curcumin, and piperine in culinary spices and herbs, as well as in essential oils and processed foods made up of these.

Alkenylbenzenes represent an important group of naturally occurring substances in plants used as food or for food production [1]. Therefore, a part of this Issue is devoted to these compounds and the associated safety issues. In this context, different aspects regarding the occurrence (e.g., in herbs and spices), toxicokinetics, toxicity, and analytics, as well as the corresponding uncertainties, were discussed in detail [2,5]. Along with this, experimental data regarding state-of-the-art analytics of alkenylbenzenes were also presented in this compilation [6].

Another complex of this Issue dwelled on the toxicology of pyrrolizidine alkaloids [7]. In this context, different studies dealing with the regulation and associated molecular processes induced by a pyrrolizidine alkaloid in experimental liver settings were also included here [8,9].

Human cancer risk is an issue of particularly high importance in the context of an exposure to potentially genotoxic and carcinogenic naturally occurring substances in plant-based food, such as alkenylbenzenes and pyrrolizidine alkaloids [2,3]. To address this topic, a review giving a detailed overview of relevant food-borne chemical carcinogens and the evidence for human cancer risk was also included in this Issue [10].

In addition, other articles published in this Issue deal with the further issues of naturally occurring compounds (e.g., curcuminoids and piperine) in different herbs and spices, such as dill, tarragon, black pepper, or turmeric [11,12,13,14]. In this context, different relevant topics were addressed in more detail, such as analytical aspects but also the current safety issues as well as potential health benefits associated with those compounds.

The scientific publications curated in this compilation certainly do not represent a comprehensive and conclusive summary of the abovementioned field of science. However, the present Special Issue offers a balanced view on the issue “Natural Compounds in Plant-Based Food”, thereby attempting to shed more light on the different relevant facets of this topic with a particular focus on the potential health effects of the ingredients present in culinary herbs and spices as well as in essential oils and other food products made of them as part of the human diet.
